# Vulnerability of tropical fish communities across depth in the central Indian Ocean

**DOI:** 10.1111/cobi.70085

**Published:** 2025-07-03

**Authors:** Paris V. Stefanoudis, Nina M. de Villiers, Mariyam Shidha Afzal, Hana Amir, Farah Amjad, Aminath Shaha Hashim, Ahmed Riyaz Jauharee, Ryan Palmer, Alex D. Rogers, Mohamed Shimal, Shafiya Naeem, Mohamed Ahusan, Lucy C. Woodall

**Affiliations:** ^1^ Nekton Foundation Oxford UK; ^2^ Museum of Natural History Oxford University Oxford UK; ^3^ The Biodiversity Consultancy Cambridge UK; ^4^ Graduate School of Engineering and Science University of the Ryukyus Okinawa Japan; ^5^ Maldives Marine Research Institute Male Maldives; ^6^ School of Ocean Sciences Bangor University Bangor UK; ^7^ College of Fisheries and Ocean Sciences The Maldives National University Male Maldives; ^8^ Blue Marine Foundation London UK; ^9^ Maldives Resilient Reefs Male Maldives; ^10^ Department of Biosciences University of Exeter Exeter UK; ^11^ South African Institute for Aquatic Biodiversity Makhanda South Africa; ^12^ REV Ocean Lysaker Norway; ^13^ Ocean Census Oxford UK; ^14^ National Oceanography Centre Southampton UK

**Keywords:** bathyal zone, functional diversity, Maldives, mesophotic coral ecosystems, rariphotic zone, resilience, shallow‐water coral reefs, 浅水珊瑚礁, 中光层珊瑚生态系统, 暗光层, 半深海带, 马尔代夫, 恢复力, 功能多样性, arrecifes coralinos someros, ecosistemas coralinos mesofóticos, diversidad funcional, Maldivas, resiliencia, zona batial, zona rarifótica

## Abstract

Coral reefs and their fish communities below scuba diving depth (>30 m), in mesophotic coral ecosystems (MCEs) (∼30–150 m), in rariphotic (150–300 m), and in upper bathyal waters (300–500 m) are often underexplored, especially in the Indian Ocean. The paucity of data, including on the biodiversity, ecology, and vulnerability of these habitats and the communities they support, leads to their omission from most conservation and management decisions and practices. We investigated for the first time the structure and diversity (taxonomic and functional) of demersal fish communities from the central and southern atolls of the Maldives, spanning a wide bathymetric gradient of 2–500 m to better understand whether and how their vulnerability changes across depth. Abundance and biomass data from transect surveys of demersal fishes were combined with species’ trait data representing life histories to estimate a series of taxonomic‐based and functional‐based diversity metrics. Distinct fish communities occurred across the different surveyed depths, highlighting the unique biological characteristics of MCEs and deep‐sea coral habitats in the Indian Ocean. Taxonomic and functional diversity decreased as depth increased, and there was little overlap between species’ life‐history strategies. This suggests deep habitats are more vulnerable than shallow habitats to disturbance events given low levels of trait redundancy that buffer species’ trait loss. Moreover, many fishes living in MCE and deep‐sea habitats were among the most functionally unique species (e.g., sharks and rays) and all were threatened with extinction. Given the suite of pressures MCEs and deep‐sea habitats are subjected to (fishing, thermal stress, pollution), their vulnerability to disturbance, and the species of conservation concern they support, we suggest they should be considered as priorities in ongoing and future conservation and marine spatial planning initiatives in the region and globally.

## Abstract (es)

Vulnerabilidad de las comunidades de peces tropicales en diferentes profundidades del Océano Índico Central

**Resumen:**

Los arrecifes coralinos y sus comunidades de peces por debajo de la profundidad de inmersión (>30 m), en ecosistemas coralinos mesofóticos (ECM) (∼30‐150 m), en aguas rarifóticas (150‐300 m) y en aguas batiales superiores (300‐500 m) suelen estar poco explorados, sobre todo en el Océano Índico. La escasez de datos, incluidos los relativos a la biodiversidad, la ecología y la vulnerabilidad de estos hábitats y de las comunidades que albergan, causa que se omitan en la mayoría de las decisiones y prácticas de conservación y gestión. Investigamos por primera vez la estructura y diversidad (taxonómica y funcional) de las comunidades de peces demersales de los atolones centrales y meridionales de las Maldivas, abarcando un amplio gradiente batimétrico de 2‐500 m para comprender mejor si su vulnerabilidad cambia en función de la profundidad y cómo lo hace. Combinamos datos de abundancia y biomasa de transectos de peces demersales con datos de rasgos de especies que representan historias vitales para estimar una serie de métricas de diversidad taxonómicas y funcionales. Observamos comunidades de peces distintas en las diferentes profundidades estudiadas, lo que pone de relieve las características biológicas únicas de los ECM y los hábitats coralinos de aguas profundas del Océano Índico. La diversidad taxonómica y funcional disminuyó a medida que aumentó la profundidad, además de que las estrategias vitales de las especies tenían coincidieron pocas veces. Esto sugiere que los hábitats profundos son más vulnerables a las perturbaciones que los poco profundos, dados los bajos niveles de redundancia de rasgos que amortiguan la pérdida de rasgos de las especies. Además, muchos peces que vivían en hábitats ECM y de aguas profundas se encontraban entre las especies funcionalmente más singulares (por ejemplo, tiburones y rayas) y todas estaban amenazadas de extinción. Dado el conjunto de presiones a las que están sometidos los ECM y los hábitats de aguas profundas (pesca, estrés térmico, contaminación), su vulnerabilidad a las perturbaciones y las especies de interés para la conservación, sugerimos que se consideren prioritarios en las iniciativas actuales y futuras de conservación y planificación del espacio marino en la región y en el mundo.

## 中印度洋热带鱼类群落在不同水深的脆弱性

【摘要】

水深超过30米(即超过水肺潜水深度)的珊瑚礁及其鱼类群落, 包括中光层珊瑚生态系统(MCEs, 约30‐150米)、弱光带(150‐300米)以及上深海带(300‐500米), 往往尚未得到充分探索, 在印度洋地区尤其如此。关于这些生境及其支持的群落的生物多样性、生态学和脆弱性等方面数据的匮乏, 导致其在大多数保护和管理决策和实践中被忽略。我们首次调查了马尔代夫中部和南部环礁水深梯度为2‐500米的地区中, 底栖鱼类群落的结构和多样性(类群和功能), 以更好地了解这些鱼类群落的脆弱性是否以及如何随水深而变化。我们结合底栖鱼类样带调查获得的丰度和生物量数据与代表物种生活史的性状数据, 估算了一系列基于类群和功能的多样性指标。不同调查深度存在不同的鱼类群落, 表明印度洋MCE和深海珊瑚生境具有独特的生物学特征。类群和功能多样性随水深增加而减少, 且在物种生活史策略之间几乎没有重叠。这表明深海生境比浅海生境更容易受到干扰事件的影响, 因为深海生境可以缓冲物种性状丧失的性状冗余性较低。此外, 许多生活在MCE和深海生境的鱼类都具有最独特的功能(如鲨鱼和鳐鱼), 且都濒临灭绝。考虑到MCE和深海生境面临的一系列压力(捕鱼、热胁迫、污染)、易受干扰的脆弱性, 及其支持的受保护物种, 我们建议在区域和全球正在进行的和未来的保护项目和海洋空间规划项目中, 应优先保护这些地区。【翻译:胡怡思;审校:聂永刚】

## INTRODUCTION

Amid a global biodiversity crisis, humanity is in a race against time to reduce its impact on nature. There is now a global ambition to conserve, restore, and sustainably manage ecosystems and their biodiversity by 2050 through a series of goals and targets outlined in the Kunming–Montreal Global Biodiversity Framework (Convention on Biological Diversity, 2022). However, fundamental knowledge gaps on key ecosystem properties (e.g., biogeography, biodiversity) and processes (e.g., ecology, functioning) for a range of ecosystems across the globe are an impediment to their successful conservation. The problem is particularly acute for ecosystems on land and at sea that are difficult and expensive to access. Remote offshore areas fall under this category, especially those located in the Global South due to historical inequities in offshore exploration and research (Bell et al., 2023). For coral‐atoll‐rich island nations in the tropics, most existing information on marine ecosystems stems from the biodiverse shallow‐water coral reefs <20‐ to 30‐m deep (Souter et al., 2021), leaving spatially adjacent environments, including mesophotic coral ecosystems (MCEs) (∼30–150 m) (Hinderstein et al., 2010), the rariphotic zone (∼150–300 m) (Baldwin et al., 2018), and aphotic habitats in upper bathyal zone (300–1000 m), grossly understudied. This data deficiency brings large uncertainties as to the benefits these deep‐water habitats provide to human well‐being and prosperity and to their vulnerability to human and climatic disturbance; hence, most conservation efforts neglect them (Stefanoudis et al., 2022).

Trait‐based approaches use information on characteristics (e.g., morphology, behavior) to describe the trait (functional) diversity of a community (McGill et al., 2006; Violle et al., 2007) and are a useful tool for understanding community assembly rules (Bruelheide et al., 2018; Jarzyna et al., 2021), ecosystem resilience (De Battisti, 2021), or guiding global conservation efforts (Gallagher et al., 2021; Loiseau et al., 2020). In the coral reef context, there have been efforts to understand the vulnerability of faunas to disturbance (Auber et al., 2022; McLean et al., 2019; Mouillot et al., 2014; Zawada et al., 2019) because reefs face multiple stressors and are predicted to be severely negatively affected by the end of the century (Sully et al., 2022). However, very few trait‐based analyses have been specifically conducted in tropical habitats deeper than 30 m (Bongaerts et al., 2019), despite the fact these ecosystems are also subject to human‐mediated disturbances including thermal stress (Diaz, Foster, et al., 2023; Frade et al., 2018), overfishing (Jacquemont et al., 2024), and plastic pollution (Pinheiro et al., 2023).

To address this knowledge gap, we investigated the structure and diversity (taxonomic and functional) of demersal fish from the central and southern atolls of the Maldives across a wide bathymetric gradient (∼2–500 m) to better understand fish assemblage vulnerability to disturbance based on life‐history strategy traits and to determine whether such vulnerability differs across depth. Based on previously reported depth‐related decreases of reef taxonomic and functional diversity in the Indian Ocean region (Diaz, Howell, et al., 2023; Loiseau et al., 2022; Stefanoudis et al., 2023; Swanborn et al., 2022), we hypothesized that there is less trait overlap at depth and hence less buffering of species’ trait loss from disturbance (increased vulnerability). We also hypothesized that successively more limiting conditions at deeper depths (i.e., less light and food) lead to more functionally specialized and unique species, mirroring observations from other marine environments with limiting conditions (e.g., deep‐sea hydrothermal vents and cold seeps [Tunnicliffe & Cordes, 2020]).

## METHODS

### Study site

**FIGURE 1:**
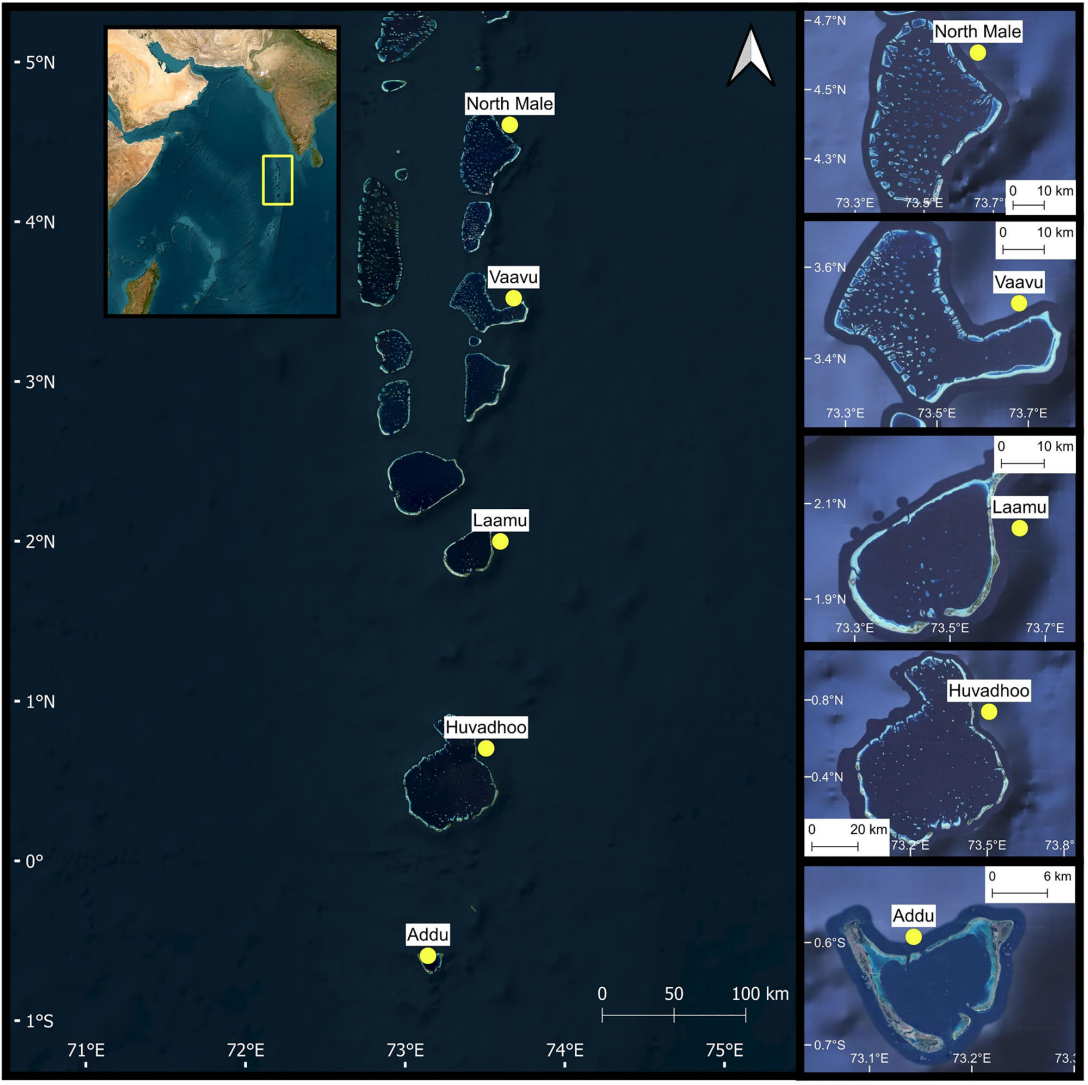
Survey sites investigated for demersal fish assemblages during the Nekton Maldives Mission.

Five coral atolls were surveyed in the Maldives (Figure 1) during The Nekton Maldives Mission onboard the *RV Odyssey* (4 September to 6 October 2022). Horizontal transect surveys were conducted across discrete depth bands (∼2–5, 10, 30, 60, 120, 250, and 500 m) through snorkeling and with a remotely operated vehicle (ROV) and manned submersibles. Each method used 2 stereo‐video systems, one facing forward and the other facing downward, to record the demersal fish and benthic communities, respectively, the former of which is the focus of this study.

### Transect surveys

At each depth, we aimed to conduct 3 replicate transect surveys that were 250‐m long. Filming was ∼1–3 m off the bottom. On a few occasions, transect surveys were not possible because of adverse weather conditions or camera malfunctions. Overall, stereo‐video footage for demersal fish was successfully collected from 104 transect surveys.

Reefs at ∼2–5 m were surveyed by snorkelers using a stereo‐video system consisting of 2 cameras (Paralenz Dive Camera+ or Paralenz Vaquita cameras). Transects followed a survey tape and moved in the direction of the prevalent current. If carried out at an outer reef, the surf break zone was avoided. Reefs at 10–30 m were surveyed using an ROV (Saab Seaeye) equipped with the same stereo‐video system, as for the snorkel transects, and lights. Reefs at 60–500 m were explored using the 3‐person Omega 3300/3 MKII class submersible (Triton) equipped with a stereo‐video system (Teledyne Bowtech L3C‐HD L3C‐HD cameras), lights (Teledyne Bowtech 6000 m‐rated V light, 20,000 lumens), and 3 lasers (Teledyne Bowtech OceanLASER‐D) arranged in a triangle 20 cm apart.

Transect survey protocols per method are detailed in Woodall et al. (2023). The number of transects per depth and location and transect length and duration per method are in Table 1. Transect‐level information is in Appendix S1.

**TABLE 1:** Summary of characteristics of transect surveys of demersal fishes in the Maldives.

Survey method	Depth (m)	No. of transects	VAA	LAA	HUV	ADD	MAL	Transect duration (min)	Transect length (m)	Distance between replicate transects
SNOR	2–5	9	2	2	3	2	–	∼13	250	∼20 m
ROV	10	15	3	3	3	3	3	∼16.5	250^a^	2–3 min of navigation
	30	15	3	3	3	3	3			
SUB	60	15	3	3	3	3	3	∼30.5	250	∼2–3 min of navigation
	120	16	3	3	4	3	3			
	250	15	3	3	3	3	3			
	500	18	5	3	4	3	3			
Total		104	22	20	23	20	18			

*Note*: Detailed information in Appendix S1.

Abbreviations: ADD, Addu; HUV, Huvadhu; LAA, Laamu; MAL, North Male; ROV, remotely operated vehicle; SNOR, snorkel; SUB, submersible; VAA, Vaavu.

^a^ Except for transects at 30 m in Vaavu that were 140‐m long because one of the cameras stopped recording.

### Data processing and analyses

Stereo‐video footage from all camera systems was calibrated and synchronized using a 3D calibration cube and CAL software 3.30 (SeaGIS Pty Ltd., Australia).

Fish identity and total length were determined and reported using EventMeasure 5.61 (SeaGIS Pty Ltd.). Where possible, all annotated fish were identified to species level based on Taquet and Diringer (2012), Fishwise Professional (www.fishwisepro.com), and FishBase (www.fishbase.org), all accessed in 2022–2023. Because it was not possible to consistently separate some species underwater and to keep consistency in identifications, we grouped together some surgeonfishes (*Ctenochaetus binotatus*, *Ctenochaetus striatus*, and *Acanthurus nigrofuscus*), butterflyfishes (*Forcipiger flavissimus* and *Forcipiger longirostris*), damselfishes (*Chromis nigrura*, and *Chromis ternatensis*; *Chromis xutha*, and *Pycnochromis agilis*; *Pomacentrus nagasakiensis*, and *Pomacentrus indicus*).

In EventMeasure, only fish positioned up to 2.5 m on either side of the stereo‐video system (transect width = 5 m) and up to 5 m in front of it were measured. Side distance restriction maintained a consistent belt along the transect, and front distance restriction prevented variation in visibility (e.g., turbidity, light intensity) from influencing data accuracy. Individuals were measured once they were as close to the cameras as possible to increase the precision of measurements. Visual surveys tend to be biased toward large, conspicuous fish, thus underestimating the densities of small, cryptobenthic fish (Galland et al., 2017). In the present survey, the minimum fish length that could reliably be detected from the stereo‐video footage was ∼1 cm. Species <2 cm comprised 0.001% of all fish counted (*n* = 50,477). Fish length measurements were converted into biomass with the equation *W* = *a* × *L^b^*, where *L* is fish length in centimeters, *W* is weight in grams, and *a* and *b* are species‐specific conversion constants from FishBase.

All fish were considered in abundance estimates, but only fish identified to species level were considered for species richness and community composition. Some fish could not be identified to species level, specifically those recorded as *Pseudanthias* at 60 m and as Anguilliformes and Ophidiiformes at 500 m, but they were nevertheless considered in biomass estimates because they were numerically common at those depths. Omitting them would have biased the results. In these cases, *a* and *b* conversion constants from the most abundant *Pseudanthias* or Anguilliformes and Ophidiiformes species observed in the same transect or location were used. Finally, in cases where the lengths of individuals could not be measured in EventMeasure, the average length of individuals in the same species from the same transect was used to calculate biomass.

A few problems with some calibrations of the submersible stereo‐video system were observed after the expedition. These were noticed when verifying data by comparing common sizes of fish observed against published information (FishBase). To correct for this, we applied a correction factor to all measurements from affected transects. We compared known distances between the triangular laser dot matrix and the values obtained from EventMeasure. Lengths were underestimated by 46%; hence, all fish lengths from the affected transects were corrected by multiplying by 1.85. To ensure that the corrected lengths were accurate, we compared the lengths of the 20 most abundant species present in this study and in a previous study in which the same methods were used (Stefanoudis et al., 2023). With this, we found no consistent length differences between the 2 studies (Appendix S2), indicating the reliability of the derived length measurements of this study.

All abundance and biomass values of each fish species per transect are in Appendix S3.

All analyses were conducted in RStudio 4.02 (RStudio Team, 2024). Where necessary, the color palette viridis from package viridis (Garnier et al., 2024) was used to produce color‐blind‐friendly graphs.

### Taxonomic diversity and composition

Differences in abundance, biomass, and species richness at different depths (fixed factor) were tested with a Kruskal–Wallis test, followed by a Mann–Whitney pairwise comparison applied with a false discovery rate correction in the package rstatix (Kassambara, 2023). Fish abundance and biomass were standardized per 100 m^2^.

Differences in fish community composition were visualized using principal coordinates ordination analysis (PCoA) on Bray–Curtis dissimilarity with square‐root‐transformed abundance data. Biomass data produced the same results and hence are not considered further. These tests were followed by permutational analysis of variance (PERMANOVA) (fixed factor depth, 9999 permutations, type III partial sums of squares, adjusted with false‐discovery rate corrections for pairwise comparisons) to identify significant differences between communities and subsequently by permutational analysis of multivariate dispersions (PERMDISP) (distances to centroids, 9999 permutations, adjusted with false discovery rate corrections for pairwise comparisons) to determine whether any of the PERMANOVA differences resulted from differences in dispersions. We did not find any consistent community patterns across sites; hence, these results are not discussed further. The PCoA, PERMANOVA, and PERMDISP were estimated using the package vegan (Oksanen et al., 2019).

### Species’ traits

We compiled data on 8 traits (age at first maturity, generation time, maximum length, minimum population doubling time [proxy for resilience], fishing vulnerability, position in the water column, preferred temperature range, vertical home range [i.e., known depth range distribution]). These traits were transformed into categorical variables to allow for use of statistics based on functional entities (i.e., distinct combinations of traits found in one or more species). Traits were chosen to broadly represent life‐history strategies, specifically population dynamics, habitat preference, and vulnerability or resilience to disturbance events, and were obtained from FishBase and occasionally were based on data from this study where relevant (Appendix S4). Although using traits from databases typically represents an average that does not capture the plasticity of traits in species’ populations (intraspecific variability [Raffard et al., 2019]) or within the lifespan of individuals (ontogenetic variability) (Zhao et al., 2014), it is practically impossible to obtain trait information from direct biological observations for hundreds of species, especially for those living in remote habitats, such as those in this study. Hence, use of trait databases represents standard practice across all realms (Mammola et al., 2022; Martini et al., 2021; Toussaint et al., 2016). Trait information was collected at the lowest possible taxonomic level and inferred, when needed, from data available for other species in the same genus or higher taxonomic levels. We also determined species’ extinction risk status from the International Union for Conservation of Nature (IUCN) Red List of Threatened Species category (www.iucnredlist.org/ [last accessed November 2023]) and species’ commercial importance in the Maldives from Maldives General Reef and Grouper Fisheries (2018–2022) and Aquarium Fisheries (2000–2015) data. Details on modalities of selected traits, risk status, commercial importance, and trait relevance to ecological functions are available in Appendix S4.

Missing trait data and the risk status categories not evaluated and data deficient were imputed using the package missForest, a nonparametric method based on random forests set to 100 random trees (Stekhoven & Bühlmann, 2012). For this procedure, we used known trait and taxonomic information, respectively, from the study's species pool (Pimiento et al., 2023). Missing data included generation time (24 spp.), age at first maturity (20 spp.), and not evaluated or data deficient status (52 spp.). The imputation error was Normalized Root Mean Squared Error = 0 and Proportion of Falsely Classified = 0.0578.

### Functional metrics

We estimated metrics introduced in Mouillot et al. (2014) that are based on functional entities (unique trait combinations), specifically functional redundancy (mean number of species per functional entity), functional over‐redundancy (percentage of species in functional entities with more species than expected from mean functional redundancy), and functional vulnerability (percentage of functional entities with only one species) (Appendix S5). We also estimated distance‐based functional diversity metrics that consider the abundance of each species: functional richness (volume of the convex hull [trait space], which encompasses all species in the assemblage), functional dispersion (average distance to the centroid of the community trait space), functional evenness (regularity of distribution along the minimum spanning tree for the studied assemblage), functional specialization (weighted mean distance to the centroid of the global species pool [center of the trait space]), and functional uniqueness (overall isolation of a species in the total trait space) (Appendix S5). Determination of the functional specialization and uniqueness metrics (in combination with risk status) allowed estimation of the FUSE (functionally unique, specialized, and endangered) index (Pimiento et al., 2020), which identifies threatened species of particular importance for functional diversity.

To estimate these metrics, we created a multidimensional trait space by applying a PCoA to a Gower similarity matrix of the species × traits table. Highly correlated traits (Kendall's correlation coefficient >0.8) were removed prior to running the PCoA (age at first maturity was highly correlated with generation time and minimum population doubling time). The mean absolute deviation index (Maire et al., 2015) indicated that the first 4 or 5 axes represented the best‐quality interpretation of the resulting trait space.

To increase computational speed, 4 axes were used to estimate the functional diversity metrics indicated. Because the estimation of alpha (transect‐level) diversity metrics (functional richness, dispersion, and evenness) requires samples (transects) to have a higher number of species than PCoA axes of the generated trait space, for these analyses only, we removed 11 transects with <5 species (2 from 120 m, 7 from 250 m, 2 from 500 m).

The traits we examined relate to species population dynamics, habitat preference, and vulnerability or resilience to disturbance; hence, our use of the term *functional* for estimated indices (e.g., functional richness) should be understood as life history strategies. We used the term *functional* to avoid discrepancies with how these indices are typically referred to in the literature. All functional‐based analyses were conducted using the package mFD (Magneville et al., 2022). Definitions and ecological relevance for each functional metric we used are in Appendix S5. Finally, we used the package psych (Revelle, 2015) to estimate the correlation between taxonomic and functional metrics with Kendall's correlation coefficient.

### Citation patterns

Considering the persistent gender bias in citation practices across all disciplines of science (Larivière et al., 2013), we sought to proactively consider citing studies that would help build a gender‐balanced reference list. We used the predicted gender tool in Google Chrome (https://chrome.google.com/webstore/detail/citation‐transparency/cepnbdbhabaljgecaddglhhcgajphbcf?hl=en) to identify the sex of the first and last authors. We then compared those values with the values in Ahmadia et al. (2021), who reported gender‐based authorship patterns of coral reef research outputs from 2003 to 2018. We focused on their reported values for 2010–2018.

## RESULTS

### Taxonomic diversity and composition across depths

**FIGURE 2:**
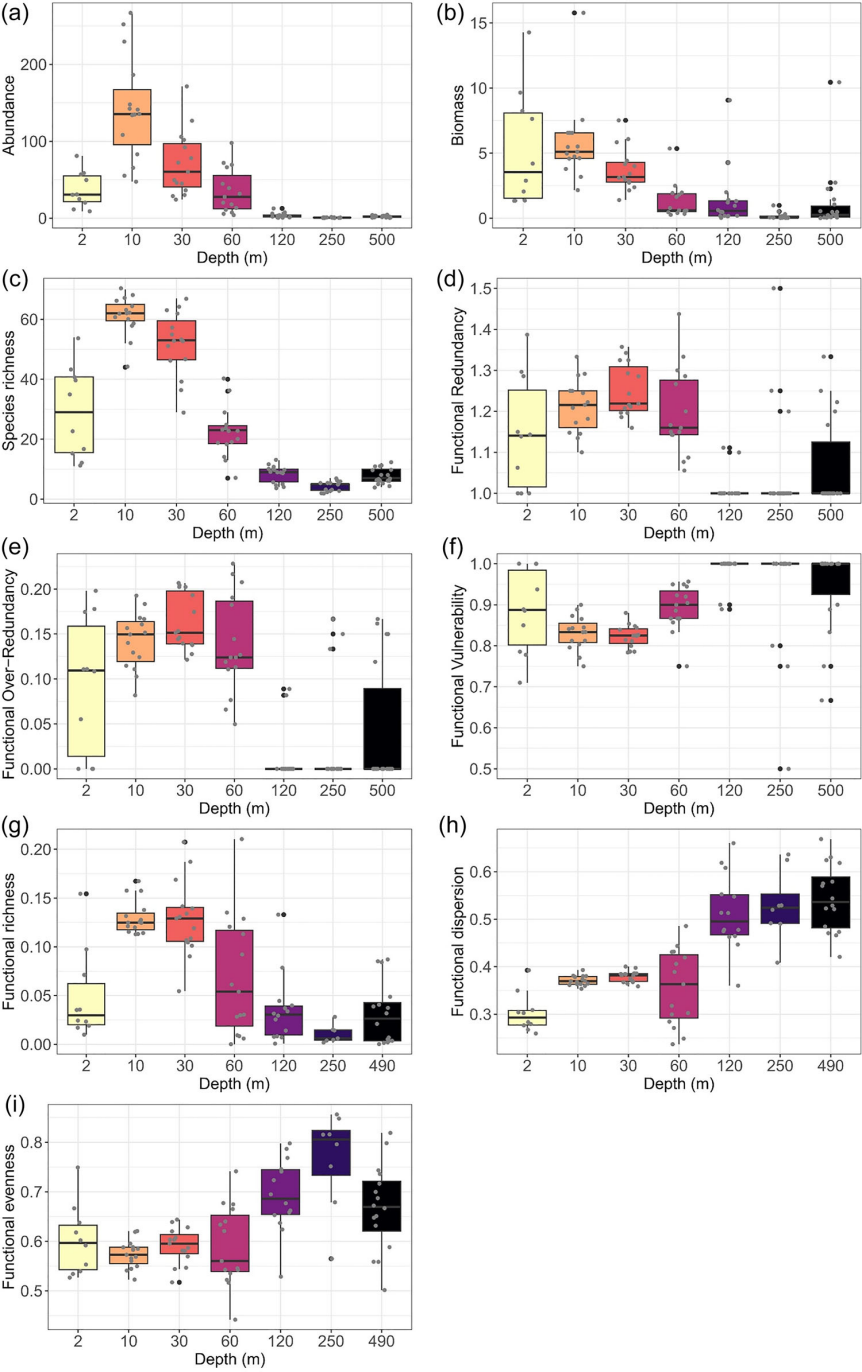
(a–c) Community metrics, (d–f) functional entities, and (g‐i) functional space metrics across depths in a survey of demersal fish assemblages in the Maldives (bar ends, 25th and 75th quantiles; horizontal lines, median; whiskers, 1.5 times the interquartile). Mann–Whitney pairwise comparisons between depths for each metric are available in Appendix S6.

A total of 309 fish species were recorded across a depth range of ∼2–500 m. Abundance and species richness peaked at 10 m, declined thereafter, and were lowest at 250 m and comparable from 120 to 500 m (Mann–Whitney, pairwise comparisons, *p* < 0.05 in all cases) (Figure 2a,c; Appendix S6). Biomass trend was broadly similar except that values were comparable from 2 to 30 m and from 60 to 120 m (Figure 2b; Appendix S6). Depth was the major driver of community composition; distinct communities were identified at each depth band (PERMANOVA pairwise comparisons, *p* < 0.05 in all cases) (Appendices S6 & S7). Homogeneity of dispersions also varied significantly across depths. It was lower in shallow water (10 and 30 m) than in other depth bands (PERMDISP, *p* < 0.05 in 18 out of 42 pairwise comparisons) (Appendix S8).

### Functional diversity and composition across depth

**FIGURE 3:**
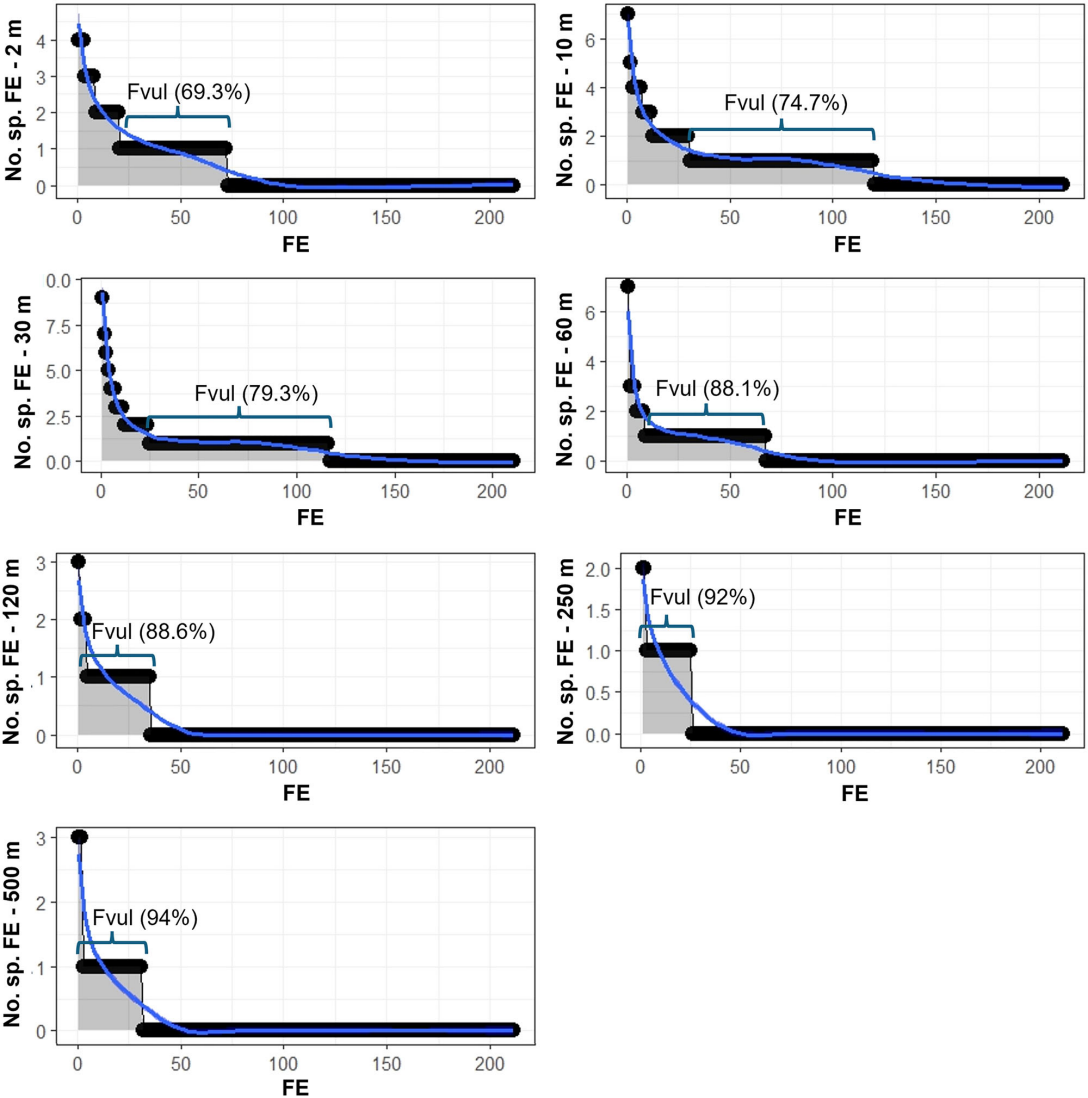
Number of fish species per functional entity (No. sp. FE) at 7 depths in the Maldives relative to the rank of FE. The percentage of functional vulnerability (Fvul) is shown above the bars and represents the percentage of functional entities at that depth represented by one species.

Functional richness peaked at 10–30 m, declined thereafter, and was lowest at 250 m (Mann–Whitney pairwise comparisons, *p* < 0.05 in all cases) (Figure 2g; Appendix S6). Functional dispersion increased as depth increased and plateaued from 250 to 500 m (Figure 2h; Appendix S6). Similarly, functional evenness increased below 120 m and peaked at 250 m (Figure 2i; Appendix S6). Functional redundancy was lower at 120–500 m than at 10–60 m but higher than at 2 m (Figure 2d; Appendix S6). Functional over‐redundancy peaked at 30 m and decreased significantly below that, especially from 120 to 250 m (Figure 2e; Appendix S6). Finally, functional vulnerability peaked at 120–500 m and was the highest at 120 m (Figure 2f; Appendix S6). The percentage of functional entities represented by one species only increased from the shallows to the deep (Figure 3).

There were strong correlations between some taxonomic‐based and functional‐based diversity metrics, most notably positive correlations between species richness and number of functional entities and functional richness (Kendall's correlation coefficient, *ρ* = 0.96 and *ρ* = 0.62, respectively) (Appendices S9 & S10). There were negative correlations between functional redundancy and vulnerability and over‐redundancy and vulnerability (*ρ* = −0.83 and −0.78, respectively) (Appendix S10).

### FUSE index

**FIGURE 4:**
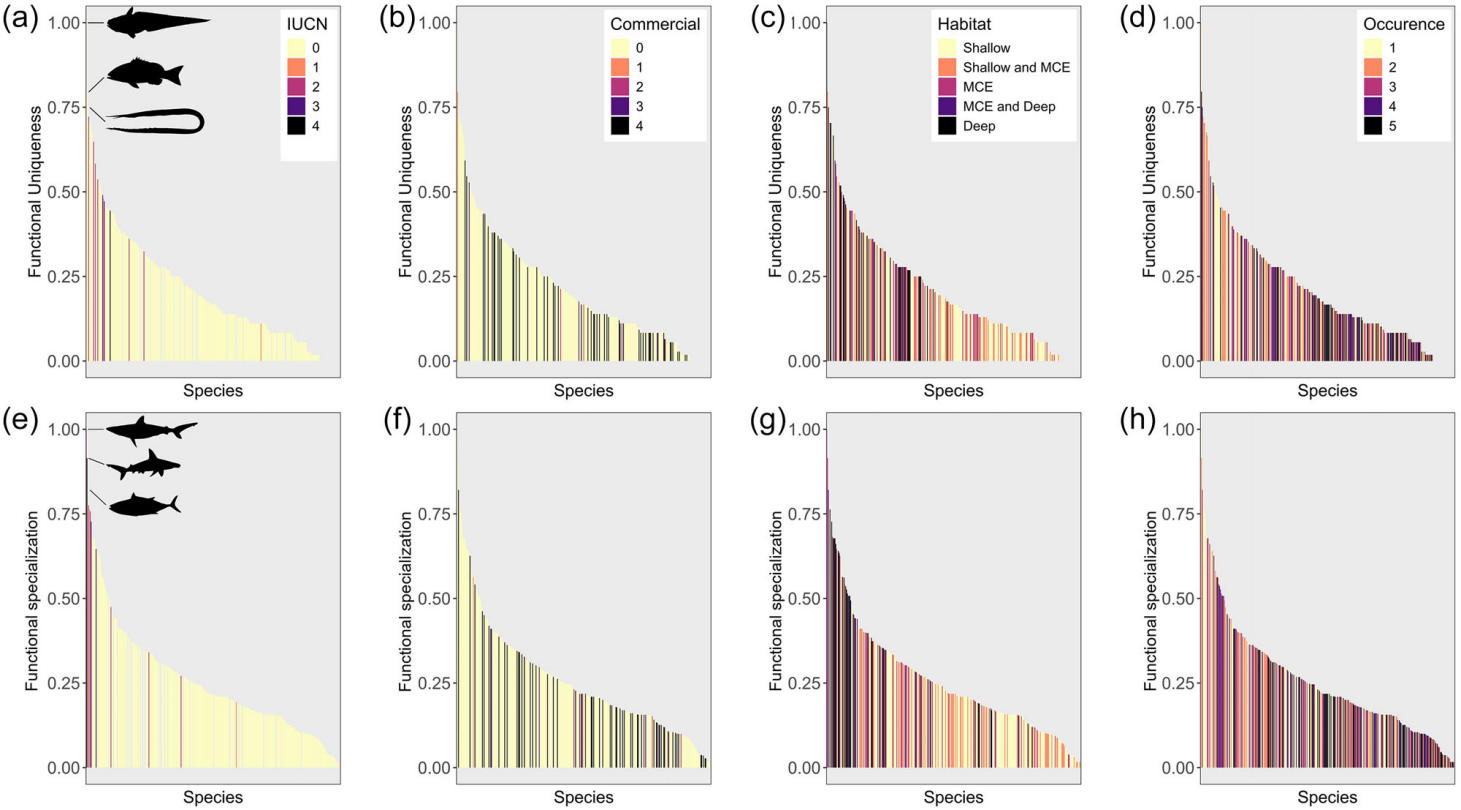
Species’ (a–d) functional uniqueness and (e–h) functional specialization relative to their (a, e) current conservation status (0, least concern; 1, near threatened; 2, vulnerable; 3, endangered; 4, critically endangered), (b, f) commercial value (0–5) (0, no commercial importance; 5, highest commercial importance to the Maldives), (c, g) habitat, and (d, h) occurrence (bars, species; IUCN, International Union for Conservation of Nature; MCE, mesophotic coral ecosystems).

A rattail fish (*Coelorinchus* sp. 1), the 2‐spot red snapper (*Lutjanus bohar*), and a duckbill eel (*Nettastoma* sp. 1) were the 3 most functionally unique species (Figure 4a–d). Silvertip shark (*Carcharhinus albimarginatus*), scalloped hammerhead shark (*Sphyrna lewini*), and the dogtooth tuna (*Gymnosarda unicolor*) were the 3 most functionally specialized species (Figure 3e–h). Values for all species are in Appendix S11. Several of the most functionally unique and specialized species were found in MCE and deep‐sea waters (Figure 4c,g).

In terms of the FUSE index, which includes functional uniqueness, specialization, and IUCN status, scalloped hammerhead shark, shortspine spurdog (*Squalus mitsukurii*), and panther electric ray (*Torpedo panthera*) had the highest scores (Table 2; full list in Appendix S11). Of the 10 species with the highest FUSE scores, only brown‐marbled grouper (*Epinephelus fuscoguttatus*) was commercially important for the Maldives. All 10 had limited geographical distribution (i.e., encountered across 1 or 2 atolls) and low abundance (<2 individuals per 1250 m^2^), and the majority (6 of 10) were found in MCE and deep‐sea waters.

**TABLE 2:** Ten fish species with the highest functionally unique, specialized, and endangered (FUSE) score surveyed in the Maldives.

Species	Common name	FUSE	Fun	Fspe	Commercial value	IUCN category	Habitat	O	*n*
*Sphyrna lewini*	Scalloped hammerhead shark	2.56	0.44	0.91	0	CR	MCE	2	1
*Squalus mitsukurii*	Shortspine spurdog	2.06	0.49	0.73	0	EN	Deep	1	1
*Torpedo panthera*	Panther electric ray	1.92	0.44	0.65	0	EN	MCE	1	1
*Carcharhinus albimarginatus*	Silvertip shark	1.87	0.58	1.00	0	VU	MCE	1	1
*Aetobatus ocellatus*	Eagle ray	1.77	0.65	0.78	0	VU	Shallow	1	1
*Triaenodon obesus*	Whitetip reef shark	1.62	0.51	0.76	0	VU	Shallow	1	1.8
*Carcharhinus melanopterus*	Blacktip reef shark	1.56	0.72	0.48	0	VU	Shallow	2	1
*Epinephelus fuscoguttatus*	Brown‐marbled grouper	1.07	0.36	0.34	4	VU	Shallow	1	2
*Galeocerdo cuvier*	Tiger shark	0.96	0.48	0.76	0	NT	Deep	1	1
*Rhabdosargus globiceps*	White stumpnose	0.93	0.32	0.27	0	VU	MCE	1	1

Abbreviations and definitions: CR, critically endangered; deep, >150 m; EN, endangered; Fspe, functional specialization; Fun, functional uniqueness; MCE, mesophotic coral ecosystems, >30–150 m; *n*, individuals per 1250 m^2^; NT, near threatened; O, number of atolls occurred; shallows, <30 m; VU, vulnerable. Commercial value scores range from 0 to 5 (0, no commercial importance; 5, highest commercial importance to the Maldives) (Appendix S4).

### Citation patterns

It was possible to determine gender in all but one (*n* = 84) cited publication. We determined the gender of the first author in 84 publications and the gender of the last author in 81 publications (3 publications had only 1 author). A proportion >1 indicated that we overcited a particular group and vice versa. The proportion of gender‐based authorship patterns of our study compared with values reported in Ahmadia et al. (2021) (their figure 5) was as follows: woman first author, 35.71% / ∼35% = 1.02 (marginally overcited); man first author, 64.29% / ∼65% = 0.99 (marginally undercited); woman last author, 24.69% / 24.7% = 1 (same level of citation); man last author, 74.07% / ∼75.2% = 0.99 (marginally undercited); women first and last authors, 12.35% / not available; woman first author and man last author, 24.69% / not available; men first and last authors, 49.38% / not available; man first author and women last author, 13.58% / not available; and unknown categorization (this study only), 1.18%.

## DISCUSSION

### Functional diversity across depth

Mirroring taxonomic species richness (Figure 2c), functional richness decreased as depth increased (Figure 2g), similar to findings from previous studies in mesophotic and deep‐sea habitats in the region and globally (Pinheiro et al., 2023; Stefanoudis et al., 2023). The low species richness at deep depths and low dispersion of traits ultimately led to habitats from 120 to 500 m, including the lower end of MCEs and the upper bathyal zone, exhibiting lower functional redundancy (fewer species per functional entity) than at shallow depths. This makes these habitats more vulnerable to disturbance, given that a larger percentage of their functional entities are represented by only one species. The link between high functional redundancy and high resilience following disturbance seems to be a prevalent pattern across marine and terrestrial environments (Biggs et al., 2020; Cooke et al., 2019; Huang et al., 2024; Pimiento et al., 2023).

Despite the view that MCEs and deep‐sea habitats are stable environments, recent evidence suggests they are dynamic and experience natural and human‐induced disturbances (Brito‐Morales et al., 2020; Hernandez‐Agreda et al., 2022; Pinheiro et al., 2019). They are also exposed to many of the same stressors that affect shallow reef communities. For example, shallow reefs and MCEs have comparable vulnerability to severe tropical storms and intense rainfall (White et al., 2013; Wong et al., 2023). Pinheiro et al. (2023) report plastic pollution densities are higher in MCEs than in shallow reefs, although they did not quantify the impact on biological communities. Even though there is some evidence that MCEs are more protected from thermal stress than shallow reefs (Frade et al., 2018; Pérez‐Rosales et al., 2021), there have been occasions when bleaching occurred exclusively at depth and not in the shallows (Diaz, Howell, et al., 2023). There is also evidence that MCEs and deep‐sea habitats are vulnerable to species invasions; for example, lionfish (*Pterois* spp.) have been observed at >200 m in Bermuda and Honduras (Gress et al., 2017). Finally, fishing at depth, including in MCEs and deep‐sea habitats (Jacquemont et al., 2024), has increased in recent years partly in response to coastal fisheries decline (Norse et al., 2012; Sadik‐Zada et al., 2023).

Ten to 60 m, and particularly 30 m, had the greatest trait overlap (functional redundancy), meaning more species with similar life‐history strategies (Figure 2d), which provides a buffer against species’ trait loss from disturbance. The greatest functional redundancy occurred at 30 m, which probably reflected larger scale patterns in fish assemblage structure, such as the overlap of 2 distinct assemblages at boundary depths (shallow reefs and upper MCEs). These results confirm our hypothesis that vulnerability to disturbance increases as depth increases. However, even though shallow communities appeared to have more functional redundancy than deep communities, this was disproportionately represented in a few functional entities, as evidenced by their high values of functional over‐redundancy (Figure 2e), indicating that they themselves are vulnerable to disturbances that cause even small reductions in species.

Comparisons with other trait‐based studies from tropical reefs are difficult because most focused primarily on depths <50 m or on different facets of functional diversity (McLean et al., 2019, 2021; McWilliam et al., 2018). Even when the authors focused on similar depths and used the same metrics, comparisons are still not straightforward because chosen traits used to estimate functional diversity might be different or because different statistical methods were used to estimate the same metrics (e.g., convex hulls vs. probabilistic hypervolumes) (Mammola et al., 2021). Hence, comparisons must be relative to patterns rather than absolute values, unless all the abovementioned parameters are the same. From the few studies in the region that have been published and cover similar depths, it seems there is a consistent pattern of decreasing functional richness going from shallow coral reefs to MCEs (Loiseau et al., 2022) and deeper (Stefanoudis et al., 2023). Fish communities in Seychelles appear to be more resilient at greater depths, which contrasts with what we found in the Maldives. However, this is because the metrics used to infer resilience in Seychelles included dispersion of traits. With very few species present at 250 m in Seychelles, sometimes there was a single species in one transect survey and dispersion was quite low, which indicates high resilience. However, the low species diversity suggests that these communities are also vulnerable to disturbances because these could lead to loss of traits and possibly population dynamics (Stefanoudis et al., 2023).

### Contribution of species to functional diversity

The 3 species with the highest functional uniqueness and specialization values (Figure 4) exhibited extreme and uncommon trait combinations. For example, some had a large body size, long generation times, large vertical home ranges, and low to medium resilience (dogtooth tuna, silvertip shark, scalloped hammerhead shark, 2‐spot red snapper). Others had short generation times, medium body size, high temperature range, and narrow depth range (duckbill eels) or long generation times, low resilience, narrow temperature, and depth range (rattails). The depth range for these 2 undescribed rattail and duckbill eel species (*Coelorinchus* sp. 1 and *Nettastoma* sp. 1, respectively) could extend below the present depth limit of our study because they are known to inhabit deeper waters. In all cases, those species occupied distinct functional entities, meaning no other species we observed had the same combination of traits.

Sharks consistently had a high FUSE score (Table 2). Sharks play multiple roles in marine ecosystems through top‐down (e.g., predation) and bottom‐up processes (e.g., provisioning and nutrient cycling) and species interactions (e.g., competition) (Dedman et al., 2024). In coral reefs, overfishing of sharks can distort ecological pyramids through mesopredator release (Sherman et al., 2020), which leads to cascading effects down the food chain that can negatively affect herbivore density and ultimately coral fitness and recovery (Ruppert et al., 2013). However, cascading effects are equivocal and context dependent (Roff et al., 2016), and there are no data from the Maldives with which to explore these relationships. Considering the potential for diverse and hidden roles of sharks in marine ecosystems (Dedman et al., 2024) and following the precautionary principle common in global environmental protection (Cameron & Abouchar, 1991), including marine fisheries (Lauck et al., 2020), our results suggest that managing shark biodiversity is important.

Many of the top‐ranking species based on the FUSE score were threatened, mirroring previous findings on marine megafauna (Pimiento et al., 2020). These results suggest that protecting threatened species would also protect functional (trait) diversity and, potentially, ecosystem functioning, although there is still work required to link specific traits and ecosystem functions (Bellwood et al., 2019). Because many of these species occur in MCEs and deep‐sea habitats, protecting these will also aid in their conservation.

### Conservation implications

Our finding of reduced functional redundancy and, hence, potential increased vulnerability of fish communities as depth increases brings important considerations for the Maldives’ fisheries, one of the key pillars of economic activity in the country. For example, we determined the abundance and distribution of 71 fishes (23% of all observed species) that are exploited in the seafood (general reef fishes and groupers) or aquarium fisheries (Appendices S3 & S4). Other species (e.g., dark‐banded fusilier [*Pterocaesio tile*]) are among some of the most common fishes used in live bait fishing (Hemmings et al., 2023). With almost all fisheries in the Maldives targeting species found in shallow waters (Sattar et al., 2011) or larger pelagic species (e.g., tuna and billfish) found in the open ocean (Ahusan et al., 2018; Jauharee et al., 2021), there is now a trend of fishing in increasingly deeper waters (60–80 m) to source bait for the bait‐fishing industry (Jauharee et al., 2015). This often involves the use of high‐illumination (3000–5000 Watt) lights that have varying impacts on fish communities that can be species or size specific (Lomeli & Wakefield, 2012; Melli et al., 2018). More studies are needed to test this effect in the Maldives. Anecdotal evidence suggests that recreational fishing, which has developed over the past decade and of which the effects are unknown, can often reach depths of up to 500 m, where, as we found, fish communities are vulnerable to disturbance.

Apart from being vulnerable, we provided evidence that despite having lower taxonomic diversity, MCEs and deep‐sea habitats hosted several highly functionally unique and specialized species, providing increasing evidence of the underappreciated contribution of these habitats to the overall functional (trait) diversity of tropical marine ecosystems (Pinheiro et al., 2023; Stefanoudis et al., 2022). Although we did not attempt to link functional specialization and uniqueness with ecosystem functioning, there is increasing evidence from other groups and ecosystems, including forest trees (Delalandre et al., 2022) and mammals (Pavoine & Ricotta, 2023) and predatory birds and herbivorous megafauna (Cooke et al., 2020), that such species can contribute significantly to ecosystem functioning. Consequently, we suggest that where resources for marine management are limited, more effort be diverted to conserving MCEs given the unique taxonomic and functional diversity they harbor and the significant number of top‐ranking FUSE score species that reside there.

Our main finding, increased community vulnerability to disturbance resulting from reduced functional redundancy, is likely to be the case for other ecosystems with limiting environmental conditions at sea and on land, where community and trait assembly patterns are determined by habitat filtering rather than competition. For instance, there is little reported redundancy in subterranean spiders (Gibert & Deharveng, 2002), montane plants (Bricca et al., 2021), coastal lagoon macrofauna (Sigala et al., 2012), and hydrothermal vents (Alfaro‐Lucas et al., 2020). This low ecological resilience makes these habitats particularly vulnerable to perturbations and disturbance and therefore should be considered important targets for conservation. Although ecosystem representativeness is a common parameter in area‐based planning and management (Maxwell et al., 2020) and in global targets (Convention on Biological Diversity Target 3), our results suggest that further consideration should be given to prioritizing ecosystems in environments along gradients of increasingly limiting conditions.

## AUTHOR CONTRIBUTIONS

*Project conceptualization, administration, and supervision*: Paris V. Stefanoudis, Mohamed Ahusan, Shafiya Naeem, and Lucy C. Woodall. *Fieldwork data collection*: Paris V. Stefanoudis, Mohamed Ahusan, Hana Amir, Farah Amjad, Aminath Shaha Hashim, Ahmed Riyaz Jauharee, Shafiya Naeem, Ryan Palmer, Alex D. Rogers, Mohamed Shimal, and Lucy C. Woodall. *Fish image annotation*: Paris V. Stefanoudis and Nina M. de Villiers. *Taxonomic validation of identifications*: Mohamed Shimal. *Research question definition*: All authors. *Statistical analyses*: Paris V. Stefanoudis. *Data interpretation*: All authors. *Writing of original draft*: Paris V. Stefanoudis, Nina M. de Villiers, Hana Amir, and Mariyam Shidha Afzal. *Figure and table preparation*: Paris V. Stefanoudis and Nina M. de Villiers. *Review and final approval of manuscript*: All authors.

## Supporting information



Supplementary Materials

Supplementary Materials

Supplementary Materials

Supplementary Materials

Supplementary Materials

Supplementary Materials

Supplementary Materials.
