# Supplementary material for: Vulnerability of tropical fish communities across depth in the central Indian Ocean
Source: Conserv Biol. 2025 Jul 3;39(6):e70085. doi: 10.1111/cobi.70085 (PMC12658959; doi:10.1111/cobi.70085)
Supplement: Supplementary file 7 — Supplementary Materials. [file COBI-39-e70085-s005.docx]

# Supporting Information

**Vulnerability of tropical fish communities across depth in the central Indian Ocean**

Paris V. Stefanoudis*^1,2,3†^, Nina M. de Villiers^2†^, Mariyam Shidha Afzal^3^, Hana Amir^4,5^, Farah Amjad^2^, Aminath Shaha Hashim^8,9,10^, Ahmed Riyaz Jauharee^5,6^, Ryan Palmer^11^, Alex D. Rogers^12,13,14^, Mohamed Shimal^5^, Shafiya Naeem^5 ⱡ^, Mohamed Ahusan^5ⱡ^, Lucy C. Woodall^2,10 ⱡ^

^1^ Nekton Foundation, Oxford, United Kingdom; ^2^ Oxford University Museum of Natural History, Oxford, United Kingdom; ^3^ The Biodiversity Consultancy, United Kingdom; ^4^ University of the Ryukyus, Okinawa, Japan; ^5^ Maldives Marine Research Institute, Male’, Maldives; ^6^ The Maldives National University, Male’, Maldives; ^7^ Bangor University, Bangor, United Kingdom; ^8^ Blue Marine Foundation, London, United Kingdom; ^9^ Maldives Resilient Reefs, Male’, Maldives; ^10^ University of Exeter, Exeter, United Kingdom; ^11^ South African Institute for Aquatic Biodiversity, Makhanda, South Africa; ^12^ REV Ocean, Lysaker, Norway; ^13^ Ocean Census, Oxford, United Kingdom; ^14^ National Oceanography Centre, Southampton, United Kingdom

*Corresponding author: [paris.stefanoudis@oum.ox.ac.uk](mailto:paris.stefanoudis@oum.ox.ac.uk)

† These authors contributed equally; ^ⱡ^ These authors contributed equally

# Keywords

shallow-water coral reefs; mesophotic coral ecosystems; rariphotic zone; bathyal zone; Maldives; resilience; functional diversity.

# Contents

**Appendix S2**. Mann-Whitney comparisons on measured lengths of the most common (i.e., within the top 20) species observed below 60 m both in Maldives (this study) and Seychelles (Stefanoudis et al. 2023).

**Appendix S5**. Definition and ecological relevance of functional metrics used in this study.

**Appendix S7**. Principal Coordinates Analysis Ordination Plot using square-root transformed abundance data.

**Appendix S9**. Kendall’s correlations between functional entity-based and taxonomic-based diversity metrics for fish communities in Maldives.

**Appendix S10**. Kendall’s correlations between functional diversity-based and taxonomic-based diversity metrics for fish communities in Maldives. Fdis = functional dispersion, Feve = functional evenness, Fric = functional richness.

**References**.

Other supporting information material for this manuscript include the following: Appendix S1, Appendix S3-S4, Appendix S6, Appendix S8, Appendix S11.

| **Appendix S2. Mann-Whitney comparisons on measured lengths of the most common (i.e., within the top 20) species observed below 60 m both in Maldives (this study) and Seychelles (Stefanoudis et al. 2023).** Only the top N = number of individuals considered for these comparisons. Rank refers to ranking relative to all species in this study for the Maldives data, and against all species in Stefanoudis et al. 2023 for the Seychelles data. | | | | | |
| --- | --- | --- | --- | --- | --- |
|  | **Maldives** | | **Seychelles** | |  |
| **Species** | **N** | **Rank** | **N** | **Rank** | **Mann-Whitney test** |
| *Pseudanthias bimarginatus* | 484 | 3 | 1888 | 1 | <0.05; Maldives larger |
| *Pseudanthias squamipinnis* | 291 | 4 | 142 | 12 | <0.05; Maldives larger |
| *Pseudanthias cooperi* | 102 | 7 | 1705 | 2 | not sig. |
| *Symphysanodon* sp. | 76 | 12 | 315 | 7 | not sig. |
| *Odontanthias borbonius* | 72 | 13 | 148 | 10 | <0.05; Seychelles larger |
| *Acanthurus thompsoni* | 63 | 15 | 178 | 9 | <0.05; Seychelles larger |
| *Cephalopholis aurantia* | 62 | 16 | 254 | 8 | <0.05; Seychelles larger |
| *Polyipnus* spp. | 49 | 17 | 116 | 13 | <0.05; Maldives larger |

| **Appendix S5. Definition and ecological relevance of functional metrics used in this study.** Adapted from Moillot et al. 2014, Magneville et al. 2022 and Stefanoudis et al. 2023. | | |
| --- | --- | --- |
| **Metric** | **Definitions** | **Ecological relevance of high scores** |
| Functional entity richness | Number of functional entities (trait combinations) present | More trait combinations |
| Functional Redundancy | The mean number of species per functional entity. | High number of species performing similar functions |
| Functional Over-Redundancy | Percentage of species in functional entities having more species than expected from functional redundancy. | Redundancy disproportionately packed into few Fes |
| Functional Vulnerability | Percentage of functional entities having only one species | Potential decrease of functional diversity following species loss |
| Functional Richness | The size of trait space (=volume inside the convex hull) encompassing all species in the assemblage | Voluminous trait space / high trait diversity. (Potentially) more ecosystem functions / services provided |
| Functional Dispersion | Average distance to the centroid of the community trait space | Niche differentiation Relaxed ecological filters Low competition for space and resources Low redundancy Less resilience to perturbations |
| Functional Evenness | The regularity of distribution along the minimum spanning tree (i.e. the tree linking all species of the assemblage with the lowest cumulative branch length) for the studied assemblage | Most niches utilised / effective use of entire range of resources available Decreased likelihood of invasions |
| Functional Specialisation | The weighted mean distance to the centroid of the global species pool (i.e. center of the functional space) | Communities with (more) specialist taxa |
| Functional Uniqueness | Overall isolation of a species within the total trait space, which is an indicator of functional redundancy | Communities with (more) specialist taxa. Indicator of vulnerability |
| FUSE | Functionally unique, specialized, and endangered (FUSE) index that combines functional uniqueness, specialization, and global endangerment to identify threatened species of particular importance for functional diversity. | Conservation Priority |


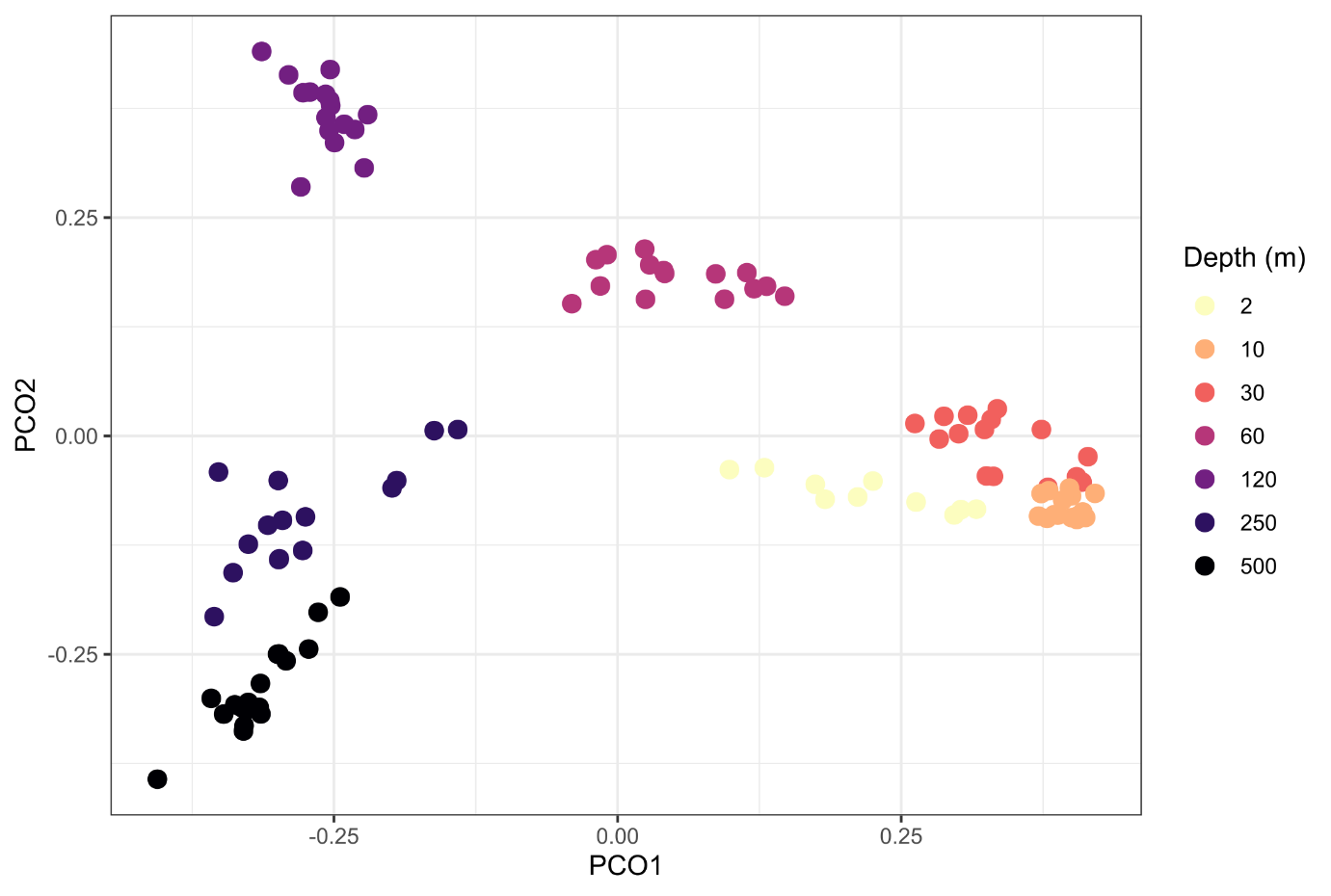


**Appendix S7. Principal Coordinates Analysis Ordination Plot using square-root transformed abundance data.**


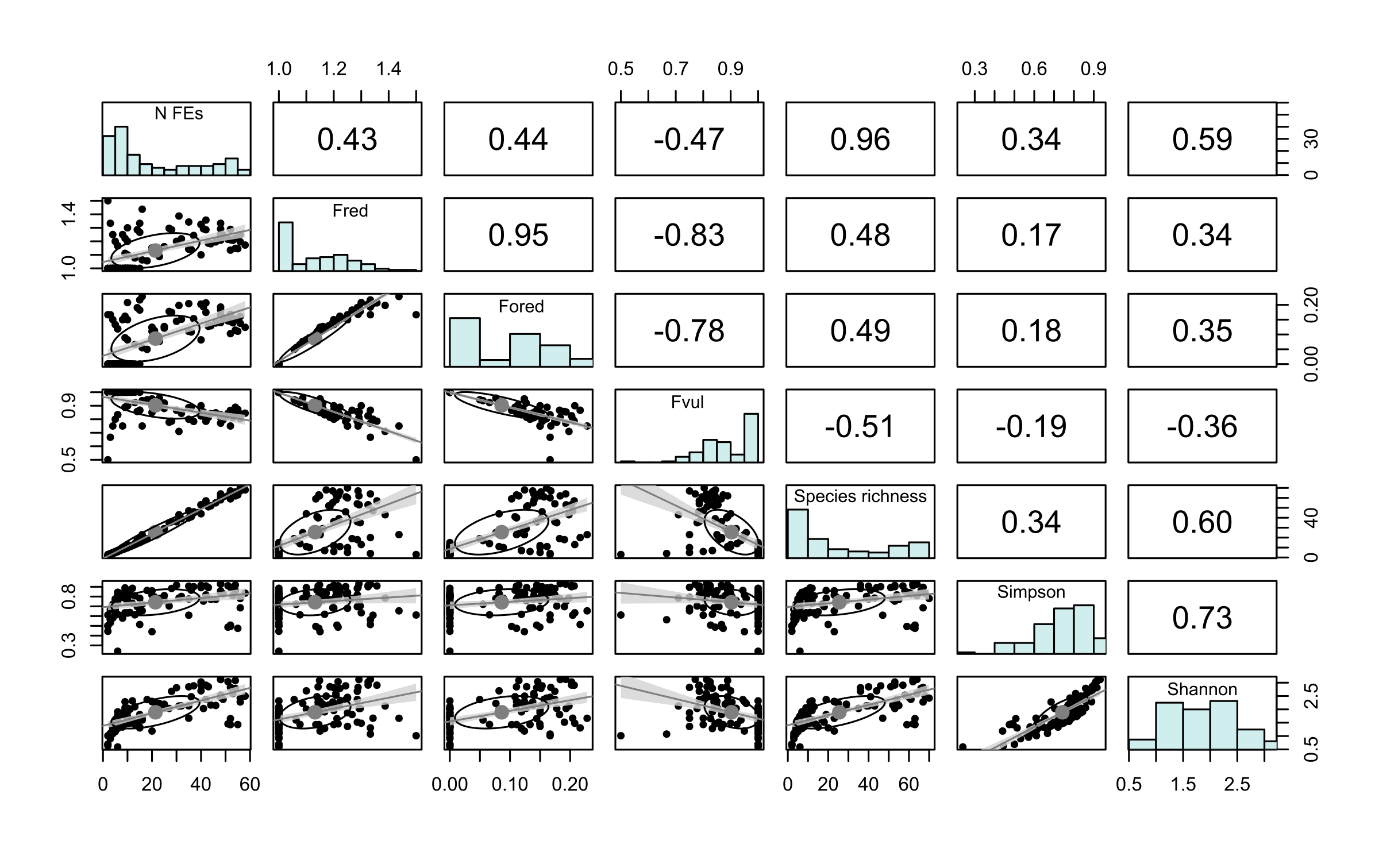


**Appendix S9. Kendall’s correlations between functional entity-based and taxonomic-based diversity metrics for fish communities in Maldives.** N FEs = number of functional entities, Fred = functional redundancy, Fored = Functional over-redundancy, Fvuln = functional vulnerability.


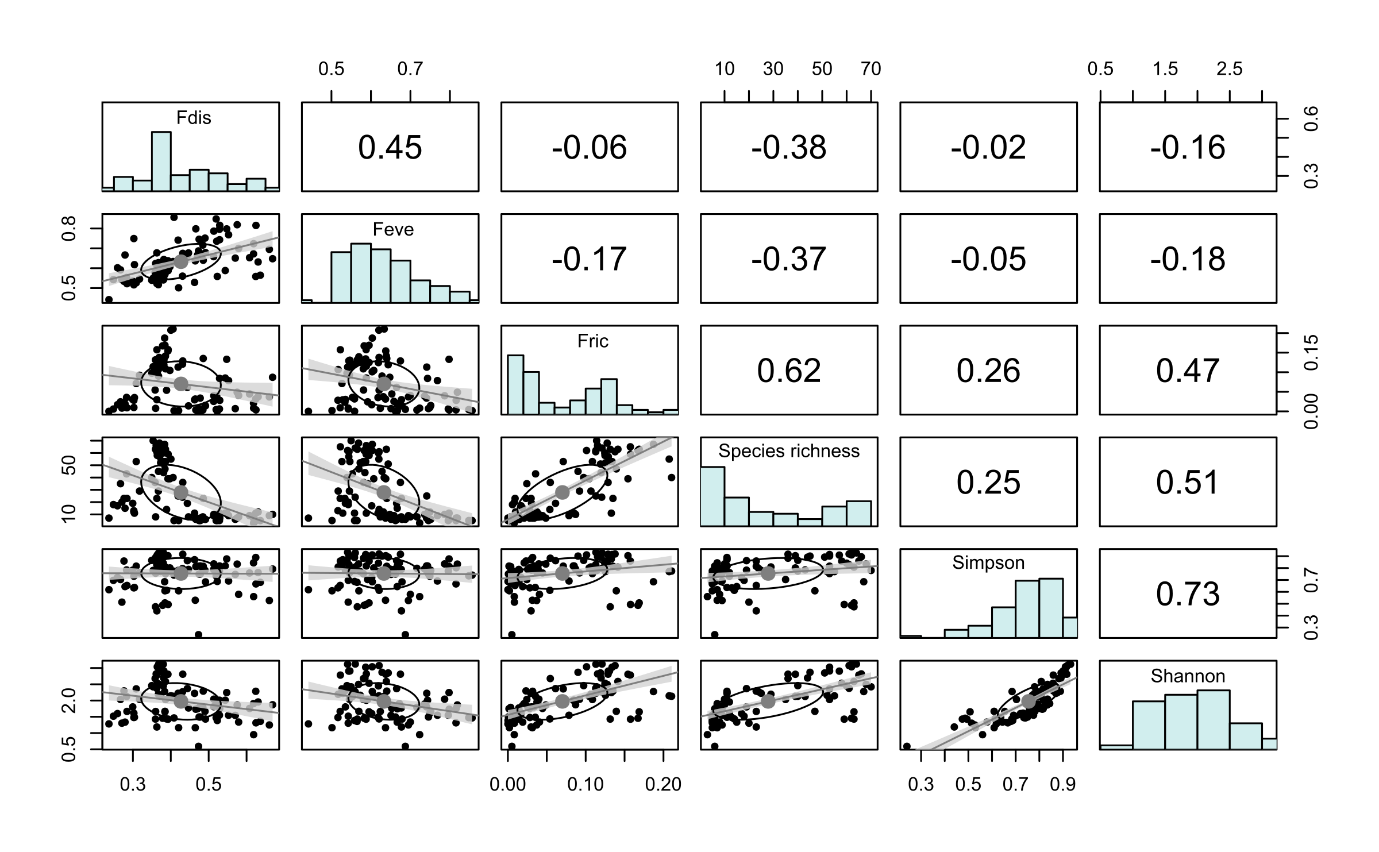


**Appendix S10. Kendall’s correlations between functional diversity-based and taxonomic-based diversity metrics for fish communities in Maldives.** Fdis = functional dispersion, Feve = functional evenness, Fric = functional richness.

**References**

Stefanoudis, P.V., Fassbender, N., Samimi-Namin, K., Adam, P.A., Ebrahim, A., Harlay, J., Koester, A., Samoilys, M., Sims, H., Swanborn, D., Talma, S., Winter, S. & Woodall, L.C. (2023). Trait-based approaches reveal that deep reef ecosystems in the Western Indian Ocean are functionally distinct. *Science of The Total Environment,* **872,** 162111. <https://doi.org/10.1016/j.scitotenv.2023.162111>
